# Non-invasive genotyping with a massively parallel sequencing panel for the detection of SNPs in HPA-axis genes

**DOI:** 10.1038/s41598-018-34223-y

**Published:** 2018-10-29

**Authors:** D. R. Gutleb, J. Ostner, O. Schülke, W. Wajjwalku, M. Sukmak, C. Roos, A. Noll

**Affiliations:** 1 Department of Behavioral Ecology, Johann-Friedrich-Blumenbach Institute for Zoology and Anthropology, University of Goettingen, Göttingen, Germany; 2 Research Group Social Evolution in Primates, German Primate Center, Leibniz Institute for Primate Research, Göttingen, Germany; 3 Leibniz Science Campus Primate Cognition, Göttingen, Germany; 4 Department of Farm Resources and Production Medicine, Faculty of Veterinary Medicine, Kasetsart University, Nakhon Pathom, Thailand; 5 Gene Bank of Primates, German Primate Center, Leibniz Institute for Primate Research, Göttingen, Germany; 6 Primate Genetics Laboratory, German Primate Center, Leibniz Institute for Primate Research, Göttingen, Germany

## Abstract

We designed a genotyping panel for the investigation of the genetic underpinnings of inter-individual differences in aggression and the physiological stress response. The panel builds on single nucleotide polymorphisms (SNPs) in genes involved in the three subsystems of the hypothalamic-pituitary-adrenal (HPA)-axis: the catecholamine, serotonin and corticoid metabolism. To promote the pipeline for use with wild animal populations, we used non-invasively collected faecal samples from a wild population of Assamese macaques (*Macaca assamensis*). We targeted loci of 46 previously reported SNPs in 21 candidate genes coding for elements of the HPA-axis and amplified and sequenced them using next-generation Illumina sequencing technology. We compared multiple bioinformatics pipelines for variant calling and variant effect prediction. Based on this strategy and the application of different quality thresholds, we identified up to 159 SNPs with different types of predicted functional effects among our natural study population. This study provides a massively parallel sequencing panel that will facilitate integrating large-scale SNP data into behavioural and physiological studies. Such a multi-faceted approach will promote understanding of flexibility and constraints of animal behaviour and hormone physiology.

## Introduction

Recent developments in molecular techniques enable researchers to include large-scale investigations of genetic impacts on behavioural or endocrine variables at reasonable costs^1,2^. In studies on humans, the investigation of genotypic influences on phenotypic characteristics revealed that inter-individual variation can be strongly affected by genotype^3–5^. For example, genotypic variation is responsible for approximately 50% of inter-individual variation in physiological stress levels and aggression^6,7^. In animal studies, however, the consideration of underlying genotype in behavioural and physiological studies is relatively understudied. Especially regarding studies on wild, non-model populations, several authors have called for a more frequent consideration of genetic impacts on animal behaviour^1,8,9^. In this study, we provide a multi-locus genotyping pipeline, based on non-invasively sampled material from a population of wild primates, facilitating future research on social and ecological factors driving variation in stress and aggression.

Modulation of the hypothalamic-pituitary-adrenal (HPA)-axis activity is an effective mechanism mediating environmental effects on the organism including its behavioural tendencies. The HPA-axis is a central physiological pathway activated in response to stress and is conserved across vertebrates^10,11^. In behavioural ecological studies, aggressive behaviour is often linked to HPA-axis activity via quantification of cortisol, the end product of this pathway (fish^12^, birds^13^, rodents^14^, ruminants^15^, cats and dogs^16,17^, primates^18,19^). Links to behaviour have been established in rats where the stimulation of brain areas responsible for aggression causes cortisol release, and similar processes are proposed for other vertebrates, including humans^20–22^. Behavioural ecological studies on aggression often assess how the expression of aggressive behaviour is related to social dominance or affected by characteristics of the competitive situation without conceptually integrating inter-individual variation due to genetic variation.

Three main metabolic circuits contribute to the HPA-axis: the serotonin, the catecholamine and the corticoid. Genes associated with these metabolic circuits have been repeatedly targeted in human clinical stress^23,24^ and aggression^4,25,26^ research. Functional polymorphisms in the genes coding for the three subsystems may lead to dysregulations in the HPA-axis pathway and a change in how the organism reacts to external stressors. The serotonin pathway involves the serotonin transporter (SLC6A4), receptor (HTR), tryptophan hydroxylase (TPH) and monoamine oxidase^27^. The neuropeptide Y (NPY) is a neurochemical that plays a protective role in stress resilience^24,28^. The catecholamine circuit (e.g. dopamine: DRD, SLC6A3, catechol-O-methyl transferase: COMT) causes general physiological changes that prepare the body for physical activity^29^. Main components of the corticoid pathway are the corticotropin-releasing hormone (CRH), CRH receptors (CRHR), the glucocorticoid receptor (NR3C1), CRH binding protein (CRHBP), corticosteroid binding globulin (SERPINA6) and the corticotropin receptor (MC2R)^30^.

For human diagnostics in the field of behavioural genetics, studies acquire large datasets via high-throughput methods such as massively parallel sequencing (i.e. next-generation sequencing)^2^. Behavioural studies on natural animal populations, however, commonly target one or a few gene loci associated with aggression and HPA-axis activity^31–33^, whereas high-throughput multi-locus approaches are rather rare but see^1,34^. Due to the high number of genes involved in physiological pathways such as the HPA-axis, the simultaneous assessment of multiple loci known to affect certain traits promises a much more comprehensive understanding of the investigated physiological and behavioural parameters^35,36^. The introduction of massively parallel sequencing technologies makes a multi-locus approach also feasible in studies on non-model species. The generated data provide high coverage of amplicons or genomes and a large and still growing body of different bioinformatics applications helps to investigate multiple loci in a fast and parallel way (e.g. Genome Analysis Toolkit – GATK^37^, SAMtools^38^ and UCSC genome browser^39^).

Here we report a massively parallel sequencing panel for the assessment of HPA-related SNPs useful for studies investigating the genetics that underlie behavioural and endocrine variation in aggression and the physiological stress response. For this purpose, we targeted loci of previously reported SNPs in 21 candidate genes associated with the HPA-axis. We provide detailed information on all steps from selection of target genes and polymorphisms, via laboratory work to the subsequent bioinformatics analyses of acquired massively parallel sequencing data. We additionally demonstrate the feasibility of application to faecal samples from wild populations, where non-invasive sampling is necessary.

## Materials and Methods

### Ethical statement

The National Research Council of Thailand (NRCT) and the Department of National Parks, Wildlife and Plant Conservation (DNP) approved (permit numbers: 0004.3/3618, 0002.3/2647, 0002/17, 0002/626, 0002/2424) the data collection at the study site in Thailand and export to Germany as part of a long-term collaboration between the University of Goettingen, the German Primate Center, the DNP and Kasetsart University Bangkok under the agreement of benefit sharing. Faecal samples were collected non-invasively. No animals were harmed or sacrificed for this study. Procedures were in accordance with the American Society of Primatologists’ (ASP) principles for the ethical treatment of non-human primates (https://www.asp.org/society/resolutions/EthicalTreatmentOfNonHumanPrimates.cfm).

### Sample collection and storage

Samples were collected at Phu Khieo Wildlife Sanctuary in north-eastern Thailand. The sanctuary is part of the 6,500 km^2^ protected Western Isaan Forest Complex. Faecal samples were collected from four groups of fully habituated and individually identifiable Assamese macaques. In total, we collected 478 faecal samples from 38 adult males and 41 adult females (1–15 per individual, Ø 6) over the course of the long-term field project between June 2006 and January 2016. 58% of the samples were collected between March 2015 and January 2016. Adult males are involved in reproduction, have fully developed testes and long canines. Females are considered as adult in the mating season that they first conceive in, dating back from observations of their first birth.

For genetic analyses ~5 g of faeces were collected immediately after defecation from the surface of the faecal sample from an identified individual. We applied the two-step storage procedure, which included the collection of faecal samples into 50 ml tubes (62.559.001, Sarstedt, Nümbrecht, North Rhine-Westphalia, Germany) containing 30 ml of 97% ethanol^40^. After storage for 24 to 36 hours, ethanol was poured off and the faecal samples were dried and stored on 30 ml silica beads (112926-00-2, Intereducation Supplies Co., Ltd., Bangkok, Thailand) in 50 ml tubes in the dark at room temperature^41^. These samples were exported to Germany within 6 months and then stored at −20 °C until DNA extraction was performed.

### SNP selection and amplicon primer design

Target regions were determined by searching the literature for SNPs in candidate genes involved in stress and aggression. The majority of target regions were chosen from literature on humans (for references see Supplementary Table S1), but we also targeted macaque and pig SNPs (for full list see Supplementary Table S1). Further, we chose only target regions located in protein-coding genes that code for receptors, enzymes and transmitter molecules associated with the HPA-axis. Targets were located both in exonic and intronic as well as untranslated regions. In total, we selected 46 target regions in 21 candidate genes. A summary about all genes that were included in the multi-locus genotyping panel can be found in Table 1, for more details about the target regions, including chromosomal position, SNPs, functional consequences and selected references see Supplementary Table S1.

**Table 1:** List of all genes included in the multi-locus genotyping panel.

HGNC gene symbol	Name	Information
AVPR1B	arginine vasopressin receptor 1B	• present in the brain as well as in the pituitary where it stimulates ACTH release • responsible for mediating the effects of vasopressin on ACTH release • inactivation reduces aggression
BDNF	brain-derived neurotrophic factor	• associated with stress vulnerability • involved in emotion processing and cognition • ‘neurotrophic hypothesis’ states that stress reduces BDNF activity resulting in decreased function within limbic brain regions
COMT	catechol-O-methyl transferase	• degrades catecholamines such as dopamine, epinephrine and norepinephrine • due to its function in catecholamine degradation and dopamine inactivation it plays a pivotal role in neuro-cerebral stress processing • essential neuropeptide for maintenance of homeostasis • functional gene variants associated with enzyme activity, cortisol levels and aggression
CRH	corticotropin-releasing hormone	• plays a crucial role in the behavioural and neuroendocrine stress response • dysregulations are linked to stress-related psychiatric disorders • experimental manipulations demonstrated that naturally occurring gene variants mediate individual variability in behavioural and physiological traits, thus determining an individual’s coping style
CRHBP	corticotropin releasing hormone binding protein	• this high-affinity protein regulates CRH availability • widely distributed throughout the body • variation in CRHBP expression influences the effectiveness of CRH in stimulating ACTH to release cortisol
CRHR1	corticotropin-releasing hormone receptor 1	• binds CRH • important for endocrine and behavioural stress response • polymorphisms predict baseline cortisol and stress-related psychotic disorders
CRHR2	corticotropin-releasing hormone receptor 2	• binds CRH in the membranes of hormone-sensitive cells
DRD3	dopamine receptor D3	• expressed in phylogenetically old regions of the brain • thus plays a role in cognitive and emotional functions • allelic variants are associated with aggression and personality
FKBP5	FK506 binding protein 5	• is a co-chaperone of the glucocorticoid receptor • together they form a complex that modulates cortisol binding affinity • thus, it modulates the HPA-axis via glucocorticoid receptor sensitivity • gene variants associated with baseline cortisol
HTR1A	serotonin receptor 1A	• binds serotonin • activation induces the secretion of hormones including cortisol, corticosterone, ACTH and oxytocin • important for stress-related information processing • effects on the cortisol stress response which are explained by differences in serotonin turnover • level of activation is associated with aggression
HTR1B	serotonin receptor 1B	• widely expressed in the central nervous system • function depends on location, in frontal cortex: postsynaptic receptor and inhibits dopamine release, in ganglia and striatum: inhibits serotonin release • gene variants associated with vulnerability for depression and anxiety
MAOA	monoamine oxidase A	• breaks down serotonin, melatonin, noradrenaline and adrenaline • therefore, it influences synaptic concentrations of these neurotransmitters • marker for antisocial behaviour • gene variants linked to aggression and impulsivity in animals and humans
MC2R	ACTH receptor	• binding of ACTH stimulates cortisol production • mutations cause familial glucocorticoid deficiency
NPY	neuropeptide Y	• most abundant neuropeptide in the mammalian brain and affects its activity • plays an important role in controlling physiological processes associated with stress, especially via emotion • released in response to stress • referred to as a protective neurochemical that mediates stress resilience
NR3C1	glucocorticoid receptor	• cortisol and other glucocorticoids bind to this receptor • expressed in almost every cell of the body • regulates gene transcription • gene variants are associated with cortisol levels and psychosocial stress and can cause hypersensitivity to glucocorticoids and a poor feedback regulation of the HPA-axis
OPRM1	opioid receptor mu 1	• high affinity for enkephalins and beta-endorphin • exists mostly presynaptically • gene variants associated with different endorphin affinities • in macaques a variant influences HPA-axis function in response to a variety of stressors
OXTR	oxytocin receptor	• presence in central nervous system • modulates stress, anxiety, social memory and sexual, aggressive and affiliative behaviours
SERPINA6	corticosteroid binding globulin	• binding and transport of glucocorticoids in mammals • assumed that polymorphisms affect glucocorticoid transport efficiency
SLC6A3	dopamine transporter	• actively removes neurotransmitters from the synaptic cleft • thereby regulates the synaptic availability of dopamine and the duration of dopaminergic neurotransmission • in macaques a variant is associated with dominance rank
SLC6A4	serotonin transporter	• regulates the serotonin re-uptake in the synaptic cleft • since effects of serotonin in the synapse are terminated by re-uptake, it is a crucial protein to regulate serotonin function in the brain • functional gene variants in humans and macaques have been associated with several behavioural phenotypes
TPH2	tryptophan hydroxylase 2	• it is the rate-limiting enzyme in the synthesis of serotonin • thus, responsible for the synthesis of serotonin in brain areas • associated with plasma cortisol

Note: For more details about the target regions, including chromosomal position, SNPs, functional consequences and selected references see Supplementary Table S1.

As genome data for our study species, the Assamese macaque (*Macaca assamensis*), is not available, we designed primers according to the genome sequence of the congeneric rhesus macaque (*Macaca mulatta*, v8.0.1). For detailed information about amplicon sequences of *Macaca mulatta* and human see Supplementary Table S1.

Primers were designed using the online-software Primer3Plus^42^. Due to DNA degradation in faecal samples, primers were designed to amplify short PCR products with a maximum of 380 bp (Ø 207 bp), with the target SNP being as central as possible. Primer annealing temperatures were between 54 and 60 °C, with a maximum difference of 2 °C for each primer pair. Primer annealing temperature was chosen as predicted by Netprimer (Premier Biosoft, Palo Alto, California, USA). Further steps of the primer design included: (i) specificity check: Primer-Blast, NCBI, ‘nr’ database for Mammalia^43^, (ii) dimerization check: Netprimer (Premier Biosoft, Palo Alto, California, USA), (iii) secondary structure check: The mfold Web Server, DNA Folding Form^44^. The designed primers were ordered from Metabion (Planegg/Steinkirchen, Bavaria, Germany). In total we designed 41 primer pairs for 46 target loci (Supplementary Table S1).

### Laboratory methods

DNA extraction was carried out with the First-DNA all-tissue Kit (D1002000, GEN-IAL GmbH, Troisdorf, North Rhine-Westphalia, Germany), following the manufacturer’s protocol for DNA extraction from faeces. The protocol included an overnight incubation at 37 °C with lysis buffer 1, lysis buffer 2 and proteinase K. After centrifugation, the supernatant was combined with lysis buffer 3, incubated at −20 °C for 5 minutes and centrifuged, followed by washing steps with 70% ethanol stored at −20 °C. Finally, DNA was eluted in 50 µl HPLC water (115333, Merck, Darmstadt, Hesse, Germany) and stored at −20 °C until further processing. All steps of the protocol were carried out with DNA LoBind Tubes (0030108051, Eppendorf AG, Hamburg, Germany). Total genomic DNA concentration was measured with a NanoDrop Spectrophotometer (ND-1000, PEQLAB Biotechnologie GmbH, Erlangen, Bavaria, Germany) and diluted to a final concentration of 100 ng/µl. 78% of the extracted faecal samples were collected between March 2015 and January 2016.

Target regions were amplified in 96-well plates (AB0600, Thermo Fisher Scientific, Waltham, Massachusetts, USA) with 1 U BioThermTaq DNA Polymerase (GC-002-5000, Genecraft, Cologne, North-Rhine Westphalia, Germany) in a 30 μl PCR mix (1 x reaction buffer, 0.16 mM for each dNTP, 0.33 μM for each primer, and 18 ng BSA, 100 ng template DNA), with the following thermocycler (Labcycler, Sensoquest, Göttingen, Lower Saxony, Germany) conditions: 2 minutes at 94 °C, 60 cycles of 30 seconds at 94 °C, 30 seconds at the appropriate annealing temperature (see Supplementary Table S1), 30 seconds at 72 °C, and 5 minutes at 72 °C. To check for PCR contamination, we ran 3 to 7 non-template controls on each 96-well plate. After amplification, aliquots were size-separated on 2% agarose gels along with a size standard (SM0241, Thermo Fisher Scientific, Waltham, Massachusetts, USA) to check for PCR performance and correct amplicon size. PCR products were then purified with Solid Phase Reverse Immobilization (SPRI) technology using 2.5x Ampure Beads (A63881, Beckman Coulter, Brea, California, USA) and again subjected to 2% agarose gel electrophoresis to control for purification performance. DNA concentration was measured with a Qubit 3.0 (Q32854, Thermo Fisher Scientific, Waltham, Massachusetts, USA). To test if our primers are target-specific, all 41 SPRI-purified amplicons of two individuals, acquired via PCR from faecal DNA extracts, were sequenced using Sanger technology. Therefore, we applied both amplification primers (3.3 pmol) and the Big Dye Cycle Sequencing Kit (433776452, Thermo Fisher Scientific, Waltham, Massachusetts, USA), and ran the reactions on an ABI 3130xl genetic analyzer (Thermo Fisher Scientific, Waltham, Massachusetts, USA). Sequence electropherograms were checked with DNA Baser (DNA Sequence Assembler v4, 2013, Heracle Biosoft S.R.L, Mioveni, Argeș, Romania) and compared with the respective target sequences of rhesus macaque and human.

For massively parallel sequencing, the amplicons from each individual were pooled in equimolar amounts to a total of 120 ng. Sequencing libraries were generated following the method described in Rohland *et al*.^45^ without uracil-DNA-glycosylase treatment based on Meyer and Kircher^46^ and Kircher *et al*.^47^. To check for performance of library preparation, we ran all libraries on an Agilent 2100 Bioanalyzer (Agilent Technologies, Santa Clara, California, USA) using the High Sensitivity DNA chip (5067-4626). Libraries were then pooled to a library mix with a final concentration of 10 nM. We added an additional step to control for quantity by running a qPCR with a 7500 Fast Real-Time PCR System (Thermo Fisher Scientific, Waltham, Massachusetts, USA) using the Bio-Rad EvaGreen Supermix (1725211, Bio-Rad, Hercules, California, USA) following the manufacturer’s recommendations and using three samples of the library mix and the concentration standards (5 nM, 10 nM, 20 nM). Reactions were run under the following conditions: 2 minutes at 50 °C, 10 minutes at 95 °C, 40 cycles of 30 seconds at 95 °C, 34 seconds at 60 °C and 30 seconds at 72 °C. Sequencing was conducted on an Illumina MiSeq sequencer (paired-end 150 bp) at the Microarray and Deep-Sequencing Core Facility, University Medical Center Goettingen, Lower Saxony, Germany.

### SNP calling

After Illumina sequencing, all produced FASTQ reads were quality-checked and trimmed with FastQC^48^ and Trimmomatic v0.36^49^. For SNP calling, we used the GATK best practices pipeline for germline SNP discovery^50^ as well as the SAMtools-bcftools-pipeline^38^. For both pipelines, all quality-checked reads were mapped against the genome of *Macaca mulatta* v8.0.1 using BWA MEM v0.7.12^51^. We followed the GATK best practice pipeline and did not mark duplicates in the bam-file, because it is not possible to distinguish duplicate reads in amplicon sequencing where the major proportion of sequences reads represents PCR duplicates^52,53^. A first variant call was carried out using GATK’s HaplotypeCaller. Recovered SNPs were filtered using the hard-filtering procedures recommended by GATK’s best practices (VariantFiltration). The following quality filter expression was applied: quality by depth smaller than 2.0 (QD < 2.0), mapping quality smaller than 40.0 (MQ < 40.0), Fisher strand (Phred-scaled p-value using Fisher’s Exact Test) more than 60.0 (FS > 60.0), mapping quality rank sum (the u-based z-approximation from the Mann-Whitney Rank Sum Test for mapping qualities) smaller than −12.5 (MQRankSum < −12.5), and read position rank sum (the u-based z-approximation from the Mann-Whitney Rank Sum Test for the distance from the end of the read for reads with the alternate allele) smaller than −8.0 (ReadPosRankSum < −8.0)^50^. Afterwards, Base Quality Score Recalibration (BQSR) was performed twice using GATK’s BaseRecalibrator and PrintReads. The final variant calling process was conducted with GATK’s HaplotypeCaller in GVCF mode. The produced GVCF-files were merged using GATK’s GenotypeGVCFs.

SAMtools v1.4 mpileup was also used to generate raw variant calls using *Macaca mulatta* v8.0.1 as reference genome. The following settings were used: -d (maximal per-file depth) set to 250, -E (recalculate BAQ), –BCF (generate genotype likelihoods in BCF format), and –output-tags set to DP [to get the DP (number of filtered reads covering the corresponding allele) tag in the output file]. Variant calling was done using bcftools call v1.4 with the following settings: –multiallelic-caller (alternative model for multiallelic and rare-variant calling)–variants-only (output variant sites only) -O v (output type: ‘v’ uncompressed VCF)^38^.

Only variants identified by both the GATK and the SAMtools pipeline lying within the ranges of the genes of interest were used for subsequent analyses. Additionally, we compared the number of variants called without a threshold for the Phred quality (QUAL) score, with QUAL set to be ≥30, and with QUAL set to be ≥100. The Phred quality score gives a logarithmically related prediction-value to the base-calling error. The higher the quality score, the higher the base call accuracy^54^.

### Variant annotation and effect prediction

SnpEff v4.3i^55^ and Variant Effect Predictor v87^56^ (VEP) were used for variant annotation and SNP effect prediction. We compared two different applications because variant prediction software can differ in their predicted effects^57^. With the VEP plugin ‘MaxEntScan’ it was possible to compare scores of the reference and mutant splice sites using a maximum entropy model and to predict splice site effects. Additionally, linkage disequilibrium scores were calculated using vcftools v0.1.14^58^ with the option ‘geno-r2’. Subsequently, all calculated annotations and effects were analysed in detail by hand.

## Results

Sanger sequencing of the 41 amplicons from two macaque individuals revealed that the applied primers are target-specific. From the 79 investigated macaque individuals, we obtained a total of 3066 amplicons, with a minimum of 34 and a maximum of 41 amplicons per individual (Ø 38.6). For 10 of the 79 individuals, the first faecal DNA extracts (3 samples collected in 2007 and 7 samples collected in 2015) did not yield any amplicons (probably due to inhibitors in faeces), however, amplicons were successfully obtained from second extracts derived from other samples from the same individuals in all cases.

After sequencing on Illumina’s MiSeq, we obtained a total of 23,604,930 reads. Around 95% of the reads exhibit a Phred score > 29. About 85% of the reads could be mapped against the reference genome of *Macaca mulatta* v8.0.1 with a mean depth of 3,219.81 and a mean mapping quality of 59.02. Detailed sequencing statistics can be found in Supplementary Tables S2 and S3.

### Variant calling

We compared different variant calling approaches in order to use only those variants reproduced by multiple pipelines. Using the complete dataset of shared variants without consideration of any filtering steps (QUAL, QD, MQ, FS, MQRankSum, and ReadPosRankSum), 70.12% of the variants called by SAMtools were also found with the GATK pipeline, whereas only 28.31% of the variants called by GATK were also found by SAMtools (Supplementary Figure S1). In sum, SAMtools called 230 SNPs, whereas GATK detected 373 SNPs. In total, 169 SNPs in 21 genes were verified using both variant calling approaches. 10 out of the 169 SNPs showed homozygosity for the alternate allele and thus represented simply a difference to the rhesus macaque genome, whereas 159 SNPs were identified as polymorphic sites within the study population. General descriptive statistics of the distribution of individuals being homozygous for the reference or alternate allele, or heterozygous, can be found in Table 2.

**Table 2:** General descriptive statisics of the detected SNPs in the investigated population of Assamese macaques.

Individuals	In percentage				In the number of individuals			
	Minimun	Maximum	Mean	Standard deviation	Minimun	Maximum	Mean	Standard deviation
homozygous for reference allele	0.00	98.73	82.60	28.00	0.00	78.00	62.65	22.88
heterozygous	0.00	56.96	10.28	15.29	0.00	45.00	7.83	11.82
homozygous for alternate allele	0.00	100.00	7.12	20.10	0.00	78.00	4.28	11.43

Using the described filtering steps without consideration of the QUAL scores, the number of detected variants by GATK decreased to 170 SNPs resulting in 144 SNPs in 20 genes shared by both callers. Using different QUAL scores as further selection steps, the number of detected polymorphisms changed again. Extracting only those variants with a QUAL score ≥ 30, GATK still detected 170 SNPs, but SAMtools called only 194 SNPs (Supplementary Figure S1). Only 140 SNPs in 20 genes were shared by both callers. With a QUAL score ≥ 100, the number of variants decreased further to 124 shared SNPs in 20 genes out of 170 SNPs detected with GATK and 162 SNPs detected with SAMtools (Supplementary Figure S1).

### Variant effect prediction

The variants detected by both the SAMtools and the GATK pipeline were used to predict possible variant effects. For this purpose, we used two applications: VEP and SnpEff. Comparing the results, almost all variants were classified as the same consequence type in both applications. The only differences are two counts of ‘5’-UTR premature start codon gain variant’ found with SnpEff, but not with VEP and 25 additional ‘intergenic variants’ found by SnpEff (Table 3). Consequently, the predicted variant effects show a high similarity between both methods concerning their impact (Table 4).

**Table 3:** Count and percent of consequence types of SNPs predicted by VEP and SnpEff.

Consequence type	VEP		SnpEff	
	Count	Percent	Count	Percent
3_prime_UTR_variant	13	2.36%	13	2.23%
5_prime_UTR_premature_ start_codon_gain_variant	0	0%	2	0.34%
5_prime_UTR_variant	6	1.09%	6	1.03%
downstream_gene_variant	20	3.62%	20	3.44%
frameshift_variant	0	0.00%	0	0.00%
intergenic_variant	11	1.99%	39	6.70%
intron_variant	260	47.10%	260	44.67%
missense_variant	62	11.23%	62	10.65%
splice_region_variant	19	3.44%	19	3.26%
synonymous_variant	105	19.02%	105	18.04%
upstream_gene_variant	56	10.14%	56	9.62%
total	552	100.00%	582	100.00%

**Table 4:** Count and percent of impact classes of SNPs predicted by VEP and SnpEff.

Consequence type	VEP		SnpEff	
	Count	Percent	Count	Percent
moderate	122	22.89%	124	22.03%
low	62	11.63%	62	11.01%
modifier	349	65.48%	377	66.96%
total	533	100%	563	100.00%

Using the VEP plugin MaxEntScan, two SNPs were found to be associated with different entropies at splice sites. These SNPs were located in the serotonin transporter (*SLC6A3*) and the neuropeptide Y (*NPY*) genes and changed entropy from 0.40 to −4.66 and 9.38 to 9.43, respectively. These SNPs were identified as ‘splice_region_variant’ and ‘intron_variant’ by VEP and SnpEff. However, not all identified ‘splice_region_variants’ caused differences in splice site entropy, as predicted by MaxEntScan. An analysis of linkage disequilibrium using vcftools revealed that 64 SNPs were linked. Linkage r2 values ranged from 0.28 to 1 (Ø 0.91).

## Discussion

Enhancing the approach of behavioural genetics and physiogenetics in wild animals would extend our knowledge of the factors that contribute to the still not completely understood variation within and between populations under natural selection pressures^8^. Behavioural studies often investigate the impacts of personality, age, sex or external factors such as social environment, group size and dominance hierarchy to explain inter-individual differences in HPA-axis related traits and probably misrepresent the amount of variation to be explained by such factors because they neglect genetic impacts^59–62^ or focus only on one or two gene variants^31–33^, but see^34,63,64^. An extended integration of genotype information in wild populations will facilitate a more comprehensive understanding of the observed phenotypic variation. For example, variant information on multiple loci can be used to generate cumulative genetic risk scores to predict individual variation^35,65^. Among the best-studied aspects of animal behaviour in the wild are behavioural and physiological reactions to social and environmental stressors^66^. Behavioural and physiological responses are tightly linked to the HPA-axis, the main physiological pathway activated in response to stressful stimuli^67^. Thus, genes coding for the components of the HPA-axis, which act in concert to maintain homeostatic balance, are important targets for the investigation of phenotypic variation in stress- and aggression-related traits.

We provide a SNP panel that may serve as a basic tool for future studies investigating the genetics of stress and aggression in behavioural and ecological studies. The offered panel and protocol enables field biologists teaming up with a laboratory to screen entire wild animal populations for multiple highly interesting target regions in a fast and parallel way. This study demonstrates that polymorphisms at purportedly functional sites exist in HPA-linked genes in natural populations. Knowing the samples from individually-recognized individuals, which is usually the case in long-term studies on wild animal groups, allows to accurately determine population frequencies of SNPs. Further, this application is transferable to other species. The HPA-axis is conserved among vertebrates^10,11^ and orthologous gene regions can be found easily for the species of interest e.g. via BLAST^43^, PSI-BLAST^68^, BLAT^69^, SSEARCH^70^ (biology.wustl.edu/gcg/ssearch.html) or HMMER3 (hmmer.org) searches. For application to other species, we recommend searching for the primer or amplicon sequences (Supplementary Table S1) using the aforementioned software and choosing the respective sequences to design taxon-specific primers. All subsequent steps can be carried out as outlined in our protocol. With small PCR product sizes, as in our case, allelic dropout is a marginal problem, but to further minimize the risk of allelic dropout we recommend multiple PCRs per sample^71^ or to perform replicates from different samples of the same individual.

Given that acquired massively parallel sequencing data hold many opportunities for further in-depth analyses, we provide detailed information on the applied bioinformatics pipelines. For example, with the help of GATK^37^ or SAMtools^38^ variants can be detected and used for subsequent high-throughput analyses concerning their functionality and possible effects (e.g. VEP^56^ and SnpEff ^55^). However, our analyses revealed that GATK called more variants than SAMtools in all conditions of the different quality thresholds and emphasize the importance of comparing pipelines and relying on validated, intersecting sets of SNPs. Further analyses of, e.g. splice site entropy (MaxEntScan) and linkage disequilibrium (vcftools), help to interpret the effects of detected polymorphisms and their potential consequences on physiological pathways.

To promote this application for studies on wild populations of non-model organisms, in which the consideration of genotype is particularly rare, we established our methods based on faecal samples. Studies on protected, free-ranging animals are often confined to the non-invasive collection of genetic material. DNA extracts from such low-quality sources contain only small amounts of host DNA^72^. The dominance of exogeneous, non-host, e.g. microbiome or food DNA, rules out a massively parallel sequencing-application on the pure DNA extracts, without prior amplification or enrichment of target regions. Sequencing of amplicons with the traditional Sanger method is time- and cost-intensive, particularly when encountering larger sets of target regions and individuals (3066 amplicons in this study). Furthermore, when two SNPs are found in one amplicon, the haplotype structure remains unknown. When applying the classical Sanger sequencing approach, elucidating haplotypes requires additional working steps, such as cloning. Here we have shown that multiple target regions can be easily covered with massively parallel amplicon sequencing from faecal DNA of larger numbers of individuals. Alternatively, target regions could be captured using synthesized or self-made capture probes^73,74^. While such methods may reach better sequencing uniformity, they are less target-specific and exhibit lower average coverage than amplicon-based technologies^75^. However, such methods could be applied to calculate additional background population structure^74^. Another important aspect, especially for studies on wild populations that are often limited to low-quality DNA samples, is that amplicon-based massively parallel sequencing methods allow processing of low-input DNA samples^75^. Further, due to the large amount of sequence reads produced, the regions of interest (amplicons) exhibit high coverage reducing the detection of false positive variants. A caveat of the study is that it is still ultimately a bottom-up approach needing a priori information to select target regions. As technology will improve in the future, top-down approaches will most likely also become an effective and economical tool for low-quality samples making more data available. These top-down approaches could be applied to generate haplotype data for a multitude of loci across the genome in a fast and parallel way, to calculate relatedness and include kinship relations in behavioural genetics approaches as well as to identify conserved genome regions or gene segments with high mutation rates in the investigated populations.

Numerous field studies have established links between non-invasive measures of HPA-axis activity and the behaviour of animals. Glucocorticoid metabolite levels, the end products of the HPA-axis, increase during reproductive challenges^19,76^, with increasing aggression given or received^76,77^ and are often related to social status^78^. Mostly lab-based studies have established links between HPA-axis activity and genetic variation at individual loci. For example, a mu-opioid receptor polymorphism is associated with cortisol and aggressive threat scores^79^ and variation in the serotonin transporter gene is associated with increased HPA-axis activity^80^ in captive primates. Progress is hampered by a lack of (1) integration of both research streams to link genetic variation to HPA-axis activity and behaviour, and (2) studies screening multiple loci involved in HPA-axis regulation at the same time. We propose that our panel can serve as a basis for general behavioural studies aiming to extend their study design on a molecular level and step into the field of behavioural genetics. The simultaneous investigation of genes and behaviour will help to achieve a more comprehensive understanding of individual animal characteristics.

## Electronic supplementary material

Supplementary Information Figure S1

Supplementary Information Table S1 - S3

## Data Availability

Massively parallel sequencing-data were submitted to the Sequence Read Archive (SRA) available via NCBI with the accession number SRP116685. Additional data are available in the Supplementary Information.
