# Supplementary material for: Non-invasive genotyping with a massively parallel sequencing panel for the detection of SNPs in HPA-axis genes
Source: Sci Rep. 2018 Oct 29;8:15944. doi: 10.1038/s41598-018-34223-y (PMC6206064; doi:10.1038/s41598-018-34223-y)
Supplement: Supplementary file 1 — Supplementary Information Figure S1 [file 41598_2018_34223_MOESM1_ESM.pdf]

Non-invasive genotyping with a massively parallel sequencing panel for the detection of SNPs in HPA-axis genes

D. R. Gutleb[1,2,3], J. Ostner[1,2,3], O. Schülke[1,2,3], W. Wajjwalku[4], M. Sukmak[4], C. Roos[5,6], A. Noll[6]

[1] Department of Behavioral Ecology, Johann-Friedrich-Blumenbach Institute for Zoology and Anthropology, University of Goettingen, Göttingen, Germany

[2] Research Group Social Evolution in Primates, German Primate Center, Leibniz Institute for Primate Research, Göttingen, Germany

[3] Leibniz ScienceCampus Primate Cognition, Göttingen, Germany

[4] Department of Farm Resources and Production Medicine, Faculty of Veterinary Medicine, Kasetsart University, Nakhon Pathom, Thailand

[5] Gene Bank of Primates, German Primate Center, Leibniz Institute for Primate Research, Göttingen, Germany

[6] Primate Genetics Laboratory, German Primate Center, Leibniz Institute for Primate Research, Göttingen, Germany

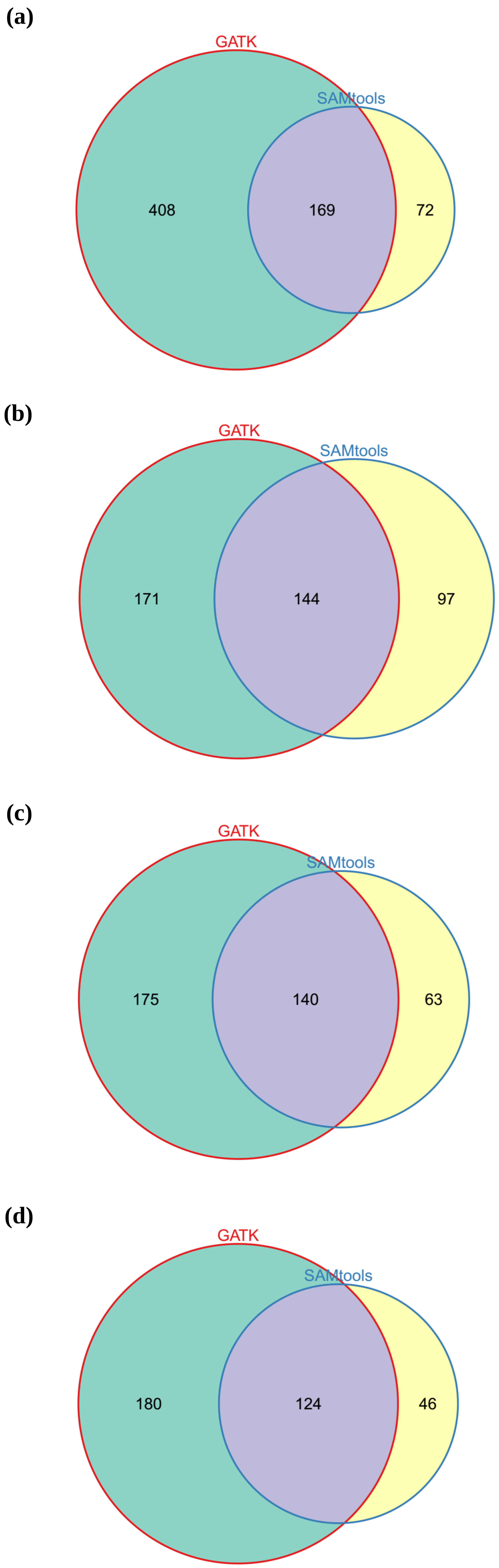

Fig. S1 Sum and overlap of called variants using GATK and SAMtools pipeline **(a)** without any filtering, **(b)** with GATK-filtering, **(c)** with GATK-filtering and Phred score (QUAL) quality filter ≥ 30, **(d)** with GATK-filtering and QUAL ≥ 100.
